# Supplementary material for: PeriTox-M, a Cell-Based Assay for Peripheral Neurotoxicity with Improved Sensitivity to Mitochondrial Inhibitors
Source: Cells. 2025 Dec 4;14(23):1929. doi: 10.3390/cells14231929 (PMC12691014; doi:10.3390/cells14231929)
Supplement: Supplementary file 1 [file cells-14-01929-s001.zip › cells-3990572-supplementary.pdf]

Supplementary information for

PeriTox-M, a cell based assay for peripheral neurotoxicity with improved sensitivity for mitochondrial inhibitors

Anna-Katharina Holzer[1], Mira Dürr[1], Selina Multrus[1], Laura Dangel[1], Viktoria Magel[1], Marcel Leist[1,2,]*

Table of Contents

Figure S1	P2	Differentiation and characterization of peripheral neurons cultured in glucose or galactose media.
Figure S2	P4	Maximal mitochondrial respiration and non-mitochondrial oxygen consumption of PNs cultured in glucose or galactose.
Figure S3	P5	Enhanced sensitivity of peripheral neurons for the complex I inhibitor tebufenpyrad and the complex III inhibitor picoxystrobin.
Figure S4	P6	Effect of the glucose-galactose-switch on the peripheral neurotoxicity of mitochondrial respiratory chain complex I inhibitors.
Figure S5	P7	Effect of the glucose-galactose-switch on the peripheral neurotoxicity of mitochondrial respiratory chain complex II inhibitors.
Figure S6	P9	Effect of the glucose-galactose-switch on the peripheral neurotoxicity of mitochondrial respiratory chain complex III inhibitors.
Figure S7	P10	Effect of the glucose-galactose-switch on the PN cell viability of mitochondrial respiratory chain complex III inhibitors.
Figure S8	P11	Effect of the glucose-galactose-switch on the peripheral neurotoxicity of proteasome inhibitors.
Figure S9	P13	Direct assessment of mitochondrial respiratory chain inhibition of test compounds.
Figure S10	P14	Comprehensive overview of tested substances revealing increased specificity of the PeriTox-M assay for neurotoxicants.
Figure S11	P16	Comparison of PeriTox-M effect concentrations to data from a central neuron-based test method.
	P17	Supplementary references

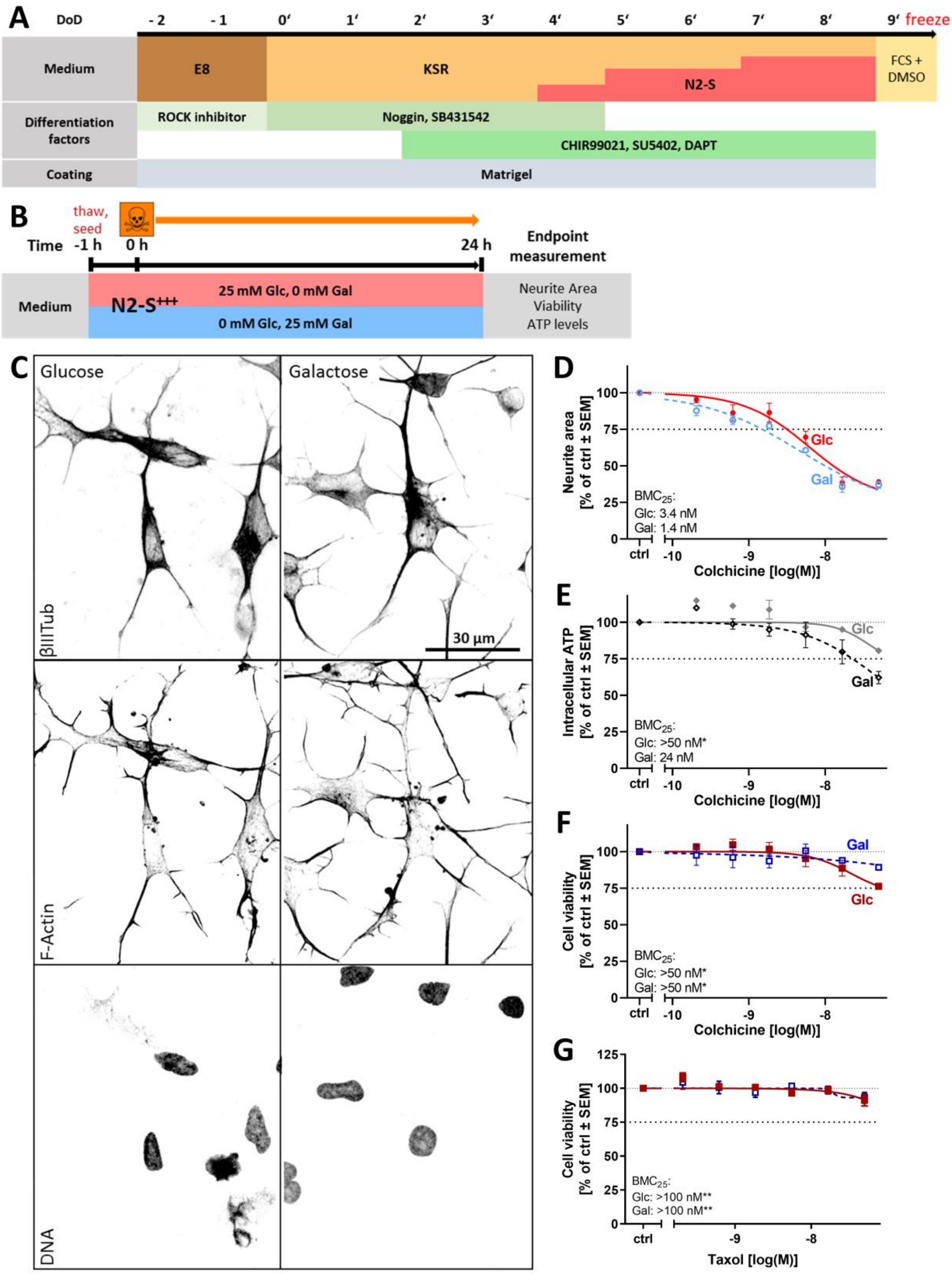

A
DoD
- 2
- 1
0′
1′
2′
3′
4′
5′
6′
7′
8′
9′ freeze
Medium
E8
KSR
N2-S
FCS + DMSO
Differentiation factors
ROCK inhibitor
Noggin, SB431542
CHIR99021, SU5402, DAPT
Coating
Matrigel
B
thaw, seed
Time -1 h
0 h
24 h
Endpoint measurement
Medium
N2-S+++
25 mM Glc, 0 mM Gal
0 mM Glc, 25 mM Gal
Neurite Area
Viability
ATP levels
C
Glucose
Galactose
βIIITub
30 µm
F-Actin
DNA
D
Neurite area [% of ctrl ± SEM]
Glc
Gal
BMC25:
Glc: 3.4 nM
Gal: 1.4 nM
Colchicine [log(M)]
E
Intracellular ATP [% of ctrl ± SEM]
Glc
Gal
BMC25:
Glc: >50 nM*
Gal: 24 nM
Colchicine [log(M)]
F
Cell viability [% of ctrl ± SEM]
Gal
Glc
BMC25:
Glc: >50 nM*
Gal: >50 nM*
Colchicine [log(M)]
G
Cell viability [% of ctrl ± SEM]
BMC25:
Glc: >100 nM**
Gal: >100 nM**
Taxol [log(M)]

Supplementary Figure S1: Differentiation and characterization of peripheral neurons cultured in glucose or galactose media.

(A) Peripheral neurons (PNs) were differentiated from the human induced pluripotent stem cell line Sigma0028 using the media, coating, and differentiation factors indicated. After two days in E8 stem cell medium, differentiation is started by switching to differentiation media containing different ratios of knockout serum replacement (KSR) and N2-S media (supplemented with noggin and the small molecule inhibitors SB431542, DAPT, CHIR99021, and SU5402). The timing of this differentiation phase is indicated by primed numbers. Cells, grown on Matrigel-coated plates, were frozen on day of differentiation (DoD) 9′ in FCS (fetal calf serum) with 10% DMSO. (B) For glucose-galactose-switch (Glc-Gal-switch) experiments, cells were thawed and seeded in N2-S medium supplemented with DAPT, CHIR99021, and SU5402 (N2-S^{+++}), containing either 25 mM Glc or 25 mM Gal as carbohydrate source. Toxicant exposure was initiated 1 h after seeding, and endpoints (neurite area, viability, ATP levels) were measured after 24 h of toxicant exposure. (C) PNs were fixed at the time of endpoint measurement and stained for the neuronal cytoskeletal marker βIII-tubulin (βIIITub) and F-actin. DNA was stained with Hoechst-33342. The signals from individual fluorescent channels are given as b/w images. The scale bar is given in the images. (D-G) PNs cultured in Glc (solid) or Gal (dashed) were exposed to the cytoskeletal drugs colchicine and taxol for 24 h: (D) The neurite area and (E) the ATP content were assessed for colchicine treated cells. (F,G) The cell viability was assessed for colchicine and taxol treated cells. The benchmark concentration at which a 25% decrease was observed (BMC25) is given in the graph. *: The BMC25 is given as the highest test concentration (HTC), since no 25% decrease was reached, but the endpoint was affected by at least 10%. **: The BMC25 is given as 2xHTC, since the endpoint was decreased by less than 10%. This data imputation is according to the data processing procedure of the PeriTox (UKN5) assay [1]. Data are means ± SEM of 3-5 biological replicates. The full data set is given in supplementary materials, ref. [2].

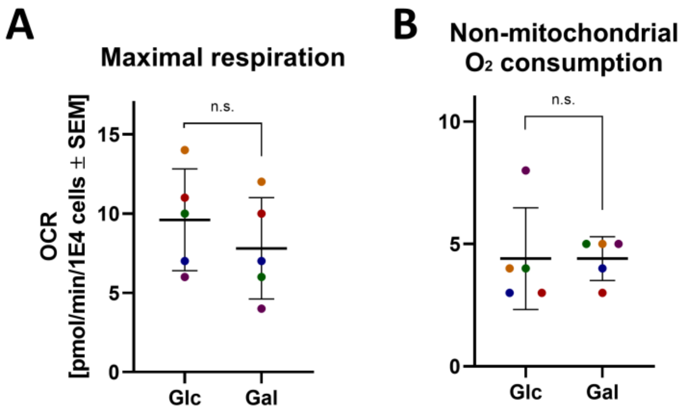

Supplementary Figure S2: Maximal mitochondrial respiration and non-mitochondrial oxygen consumption of PNs cultured in glucose or galactose.

Peripheral neurons were seeded in either glucose (Glc) or galactose (Gal) medium, and mitochondrial respiration was investigated 24 h later. (A) The oxygen consumption rate (OCR) was measured over time as detailed in the methods section and Figure 3, and the maximal respiration and non-mitochondrial O_2 consumption were calculated. Color-matched data points are derived from the same experiment. Data are means ± SEM of 5 independent experiments. Differences were tested for statistical significance using an unpaired, parametric t-test with Welch’s correction. n.s., not significant.

	Tebufenpyrad BMC_{25}(NA) [nM]			Picoxystrobin BMC_{25}(NA) [nM]		
	V	NA	ATP	V	NA	ATP
Glucose	20,000	12,000	18,000	31,000	5700	3900
Galactose	390	55	51	2500	570	400
BMC_{25}(Glc/Gal)	50	220	350	12	10	10

Supplementary Figure S3: Enhanced sensitivity of peripheral neurons for the complex I inhibitor tebufenpyrad and the complex III inhibitor picoxystrobin.

PNs were used and measured as in Figure 4. Cells were treated for 24 h with tebufenpyrad (left) or picoxystrobin (right). Based on the concentration-response-data shown in Figure 4, the benchmark concentration at which a parameter was decreased by 25% (BMC_{25}) was determined, and the quantitative summary was tabulated. The full data set based on which the BMC_{25} values were determined, is compiled in supplementary materials, ref. [2]).

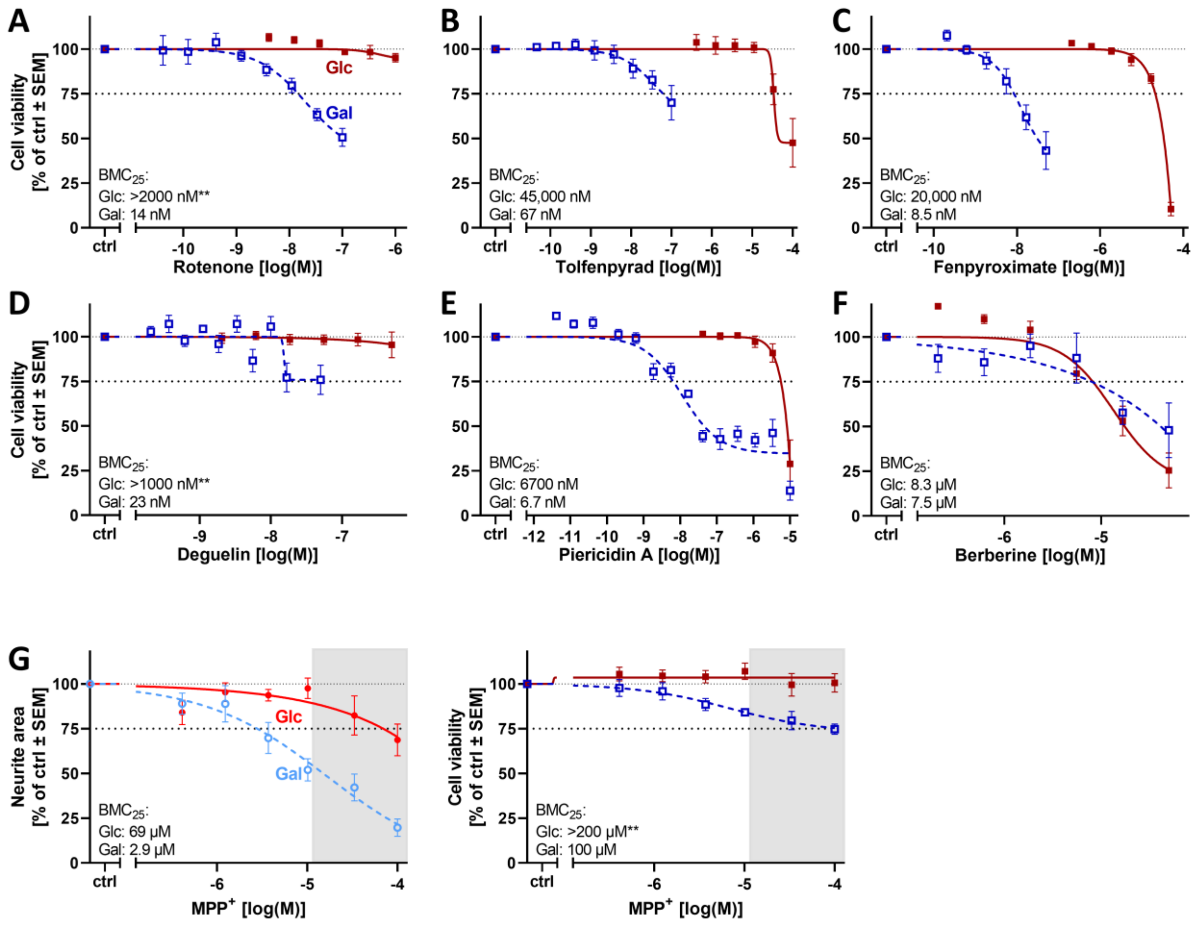

Supplementary Figure S4: Effect of the glucose-galactose-switch on the peripheral neurotoxicity of mitochondrial respiratory chain complex I inhibitors.

Various inhibitors of the mitochondrial respiratory chain complex I (cI) were tested for their neurotoxic potential as in Figure 5. The cells were treated for 24 h with cI inhibitors and the cell viability was assessed via calcein-AM/Hoechst-33342 live staining (A-F). (G) The complex I inhibitor MPP^+ was assessed for its effects on neurite area (left) and cell viability (right) with a highest test concentration of 100 µM. However, the relevant concentrations *in vivo* are known to be <10 µM [3, 4]. Therefore, the effects detected at concentrations >10 µM (gray area) are considered to be less specific. The BMC_{25} is given for culture in glucose (Glc) or galactose (Gal). Data points are given in percent of the control group (ctrl, 0.1% DMSO) and are means of 3-5 independent biological replicates ± SEM (see supplementary materials, ref. [2], for full data set). ** indicates that no effect (<10%) was measured.

.

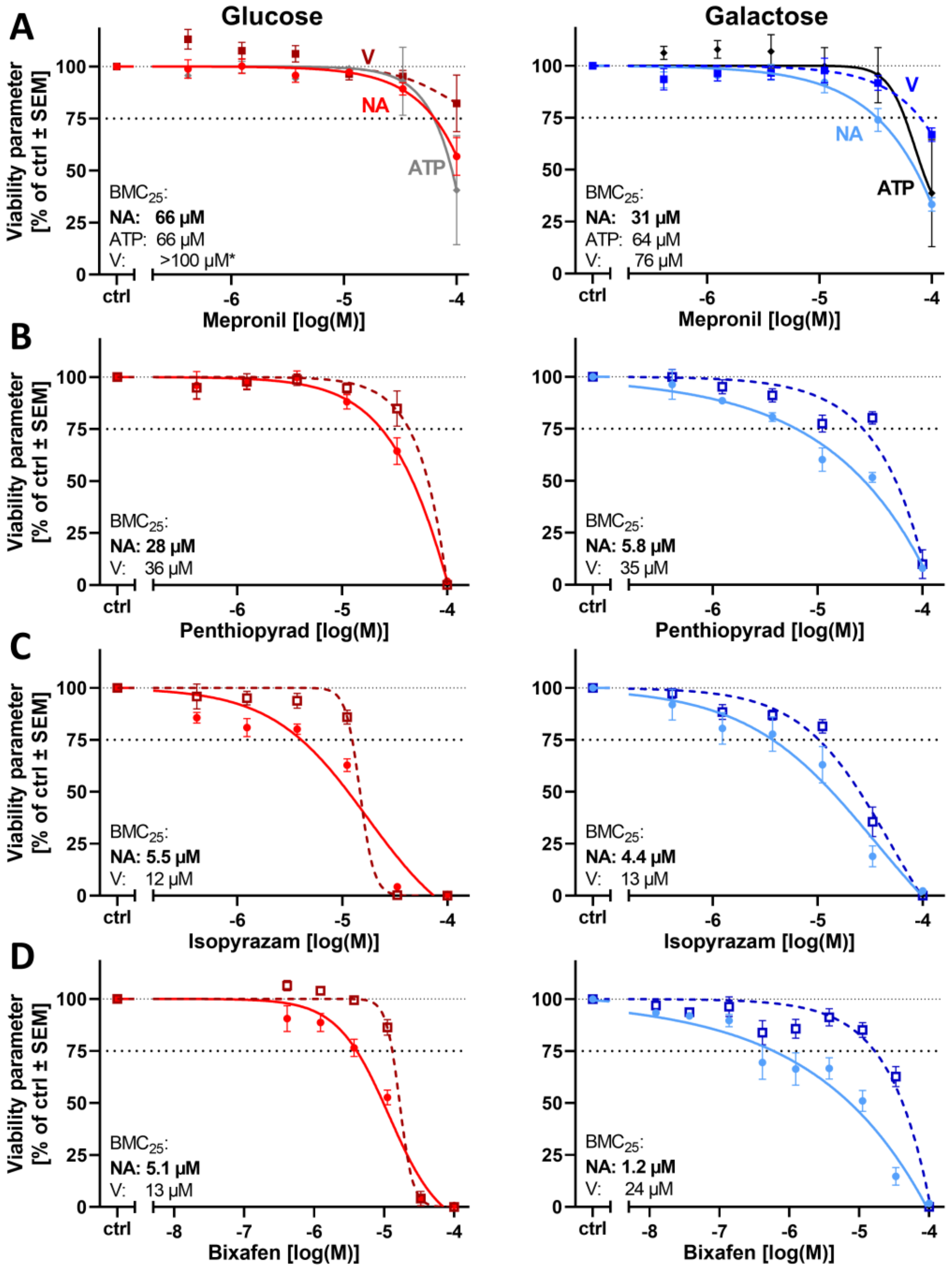

Supplementary Figure S5: Effect of the glucose-galactose-switch on the peripheral neurotoxicity of mitochondrial respiratory chain complex II inhibitors.

Various inhibitors of the mitochondrial respiratory chain complex II (cII) were tested for their neurotoxic potential as in Figure 6. PNs were cultured in either glucose (left) or galactose (right). The cells were treated for 24 h with cII inhibitors (A-D). The cell viability (V) was assessed via calcein-AM/Hoechst-33342 live staining, and the relative number of viable cells (normalized to the total cell count) is given (squares, dashed line). Based on the same imaging data, the total neurite area (NA) was determined (circles, solid line). In parallel, the ATP content was measured on the same assay plate (diamonds, solid line). The benchmark concentration at which a parameter was decreased by 25% (BMC_{25}) is given for each condition in the respective graph. Data points are given in percent of the control group (ctrl, 0.1% DMSO) and are means

of 3-5 independent biological replicates ± SEM (see supplementary materials, ref. [2], for full data set). * indicates that the BMC_{25} could not be determined in the tested concentration range, but at least 10% effect were measured.

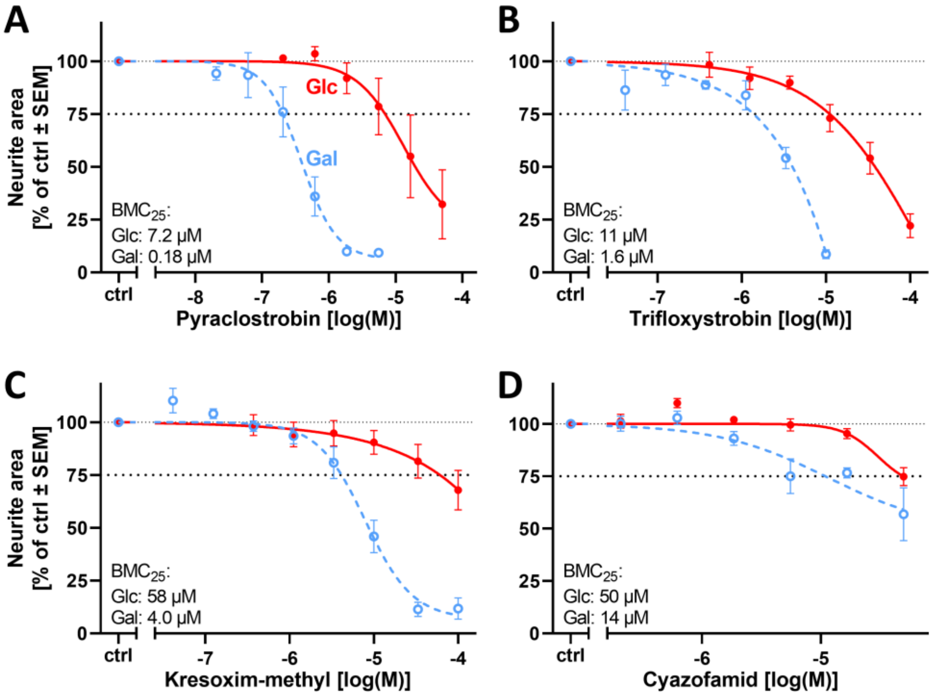

Supplementary Figure S6: Effect of the glucose-galactose-switch on the peripheral neurotoxicity of mitochondrial respiratory chain complex III inhibitors.

Various inhibitors of the mitochondrial respiratory chain complex III were tested for their neurotoxic potential as in Figure 8. Cells were treated in either glucose (Glc, solid) or galactose (Gal, dashed) for 24 h with cIII inhibitors (A-D). The neurite area was assessed via calcein-AM/Hoechst-33342 live staining. The benchmark concentration at which a parameter was decreased by 25% (BMC_{25}) is given for each condition in the respective graph. Respective viability data are given in Figure S7. Data points are given in percent of the control group (ctrl, 0.1% DMSO) and are means of 3-4 independent biological replicates ± SEM (see supplementary materials, ref. [2], for full data set).

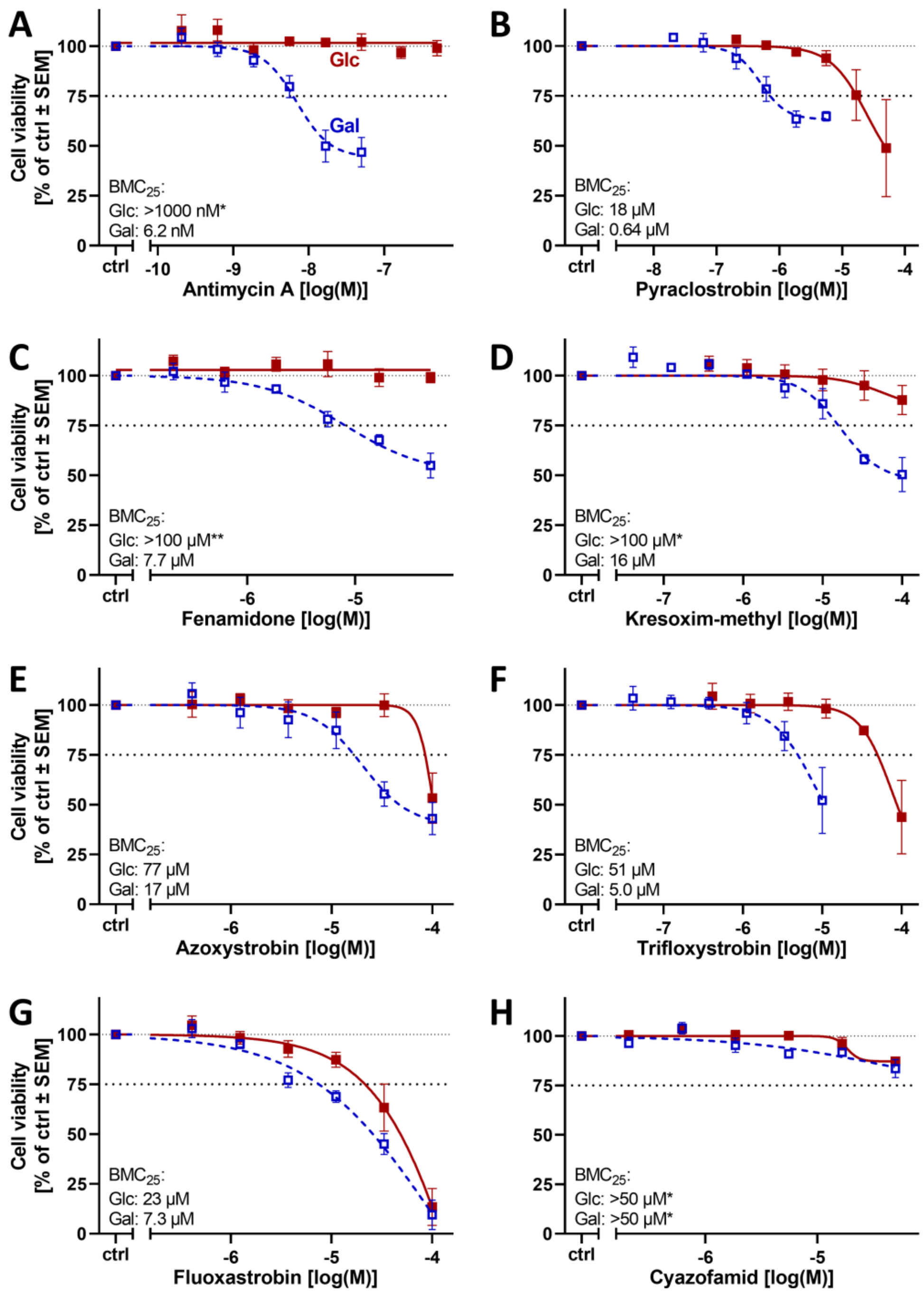

Supplementary Figure S7: Effect of the glucose-galactose-switch on the PN cell viability of mitochondrial respiratory chain complex III inhibitors.

Various inhibitors of the mitochondrial respiratory chain complex III were tested for their neurotoxic potential as in Figure 8. PNs were treated in either glucose (Glc, solid) or galactose (Gal, dashed) for 24 h with cIII inhibitors (A-H). The cell viability was assessed after calcein-AM/Hoechst-33342 live staining by high content imaging. The BMC_{25} is given for each condition in the respective graph. Data points are given in percent of the control group (ctrl, 0.1% DMSO) and are means of 3-5 independent biological replicates ± SEM (see supplementary materials, ref. [2], for full data set).

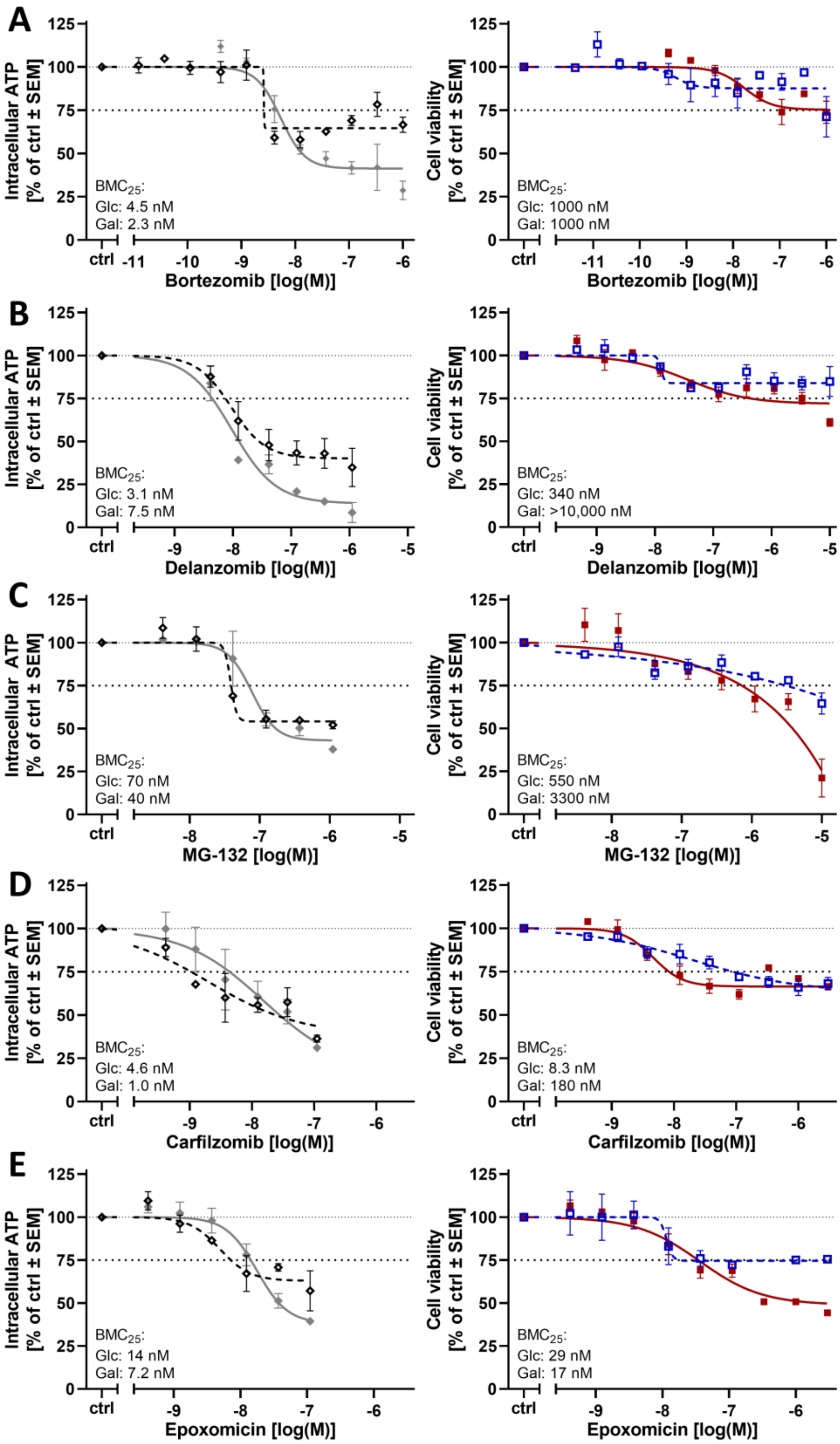
A
Intracellular ATP [% of ctrl ± SEM]
BMC25:
Glc: 4.5 nM
Gal: 2.3 nM
Bortezomib [log(M)]
Cell viability [% of ctrl ± SEM]
BMC25:
Glc: 1000 nM
Gal: 1000 nM
Bortezomib [log(M)]
B
Intracellular ATP [% of ctrl ± SEM]
BMC25:
Glc: 3.1 nM
Gal: 7.5 nM
Delanzomib [log(M)]
Cell viability [% of ctrl ± SEM]
BMC25:
Glc: 340 nM
Gal: >10,000 nM
Delanzomib [log(M)]
C
Intracellular ATP [% of ctrl ± SEM]
BMC25:
Glc: 70 nM
Gal: 40 nM
MG-132 [log(M)]
Cell viability [% of ctrl ± SEM]
BMC25:
Glc: 550 nM
Gal: 3300 nM
MG-132 [log(M)]
D
Intracellular ATP [% of ctrl ± SEM]
BMC25:
Glc: 4.6 nM
Gal: 1.0 nM
Carfilzomib [log(M)]
Cell viability [% of ctrl ± SEM]
BMC25:
Glc: 8.3 nM
Gal: 180 nM
Carfilzomib [log(M)]
E
Intracellular ATP [% of ctrl ± SEM]
BMC25:
Glc: 14 nM
Gal: 7.2 nM
Epoxomicin [log(M)]
Cell viability [% of ctrl ± SEM]
BMC25:
Glc: 29 nM
Gal: 17 nM
Epoxomicin [log(M)]

Supplementary Figure S8: Effect of the glucose-galactose-switch on the peripheral neurotoxicity of proteasome inhibitors.

Various proteasome inhibitors were tested for their neurotoxic potential. PNs were treated in either glucose (Glc, solid) or galactose (Gal, dashed) for 24 h with proteasome inhibitors as in Figure 9 (A-E). The ATP content (left) was measured and the cell viability (V) (right) was assessed after calcein-AM/Hoechst-33342 live staining by high content imaging. The BMC_{25} is given for both endpoints for each condition. Data points are given in percent of the control group (ctrl, 0.1% DMSO) and are means of 3-5 independent biological replicates ± SEM (see supplementary materials, ref. [2], for full data set).

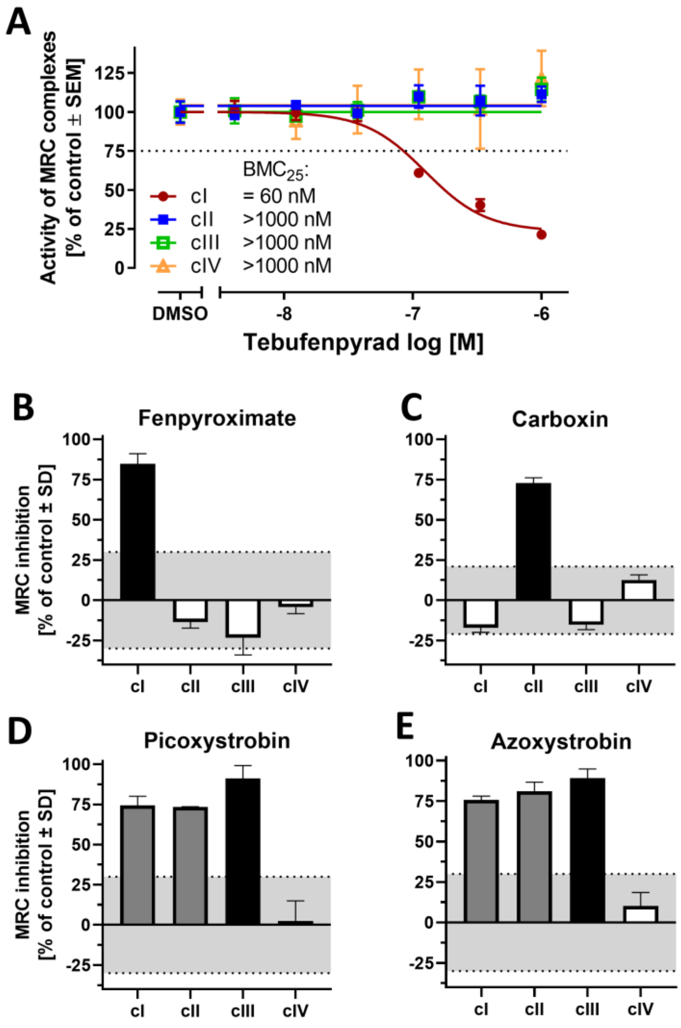

Supplementary Figure S9: Direct assessment of mitochondrial respiratory chain inhibition of test compounds.

A previously established neuronal mitochondrial respiratory chain (MRC) complex inhibition assay was adapted to peripheral neurons (PNs). PNs at the neurite outgrowth state were seeded in glucose medium. After 24 h, cells and their outer mitochondrial membranes (but not the inner mitochondrial membrane) were permeabilized. Their oxygen consumption rate (OCR) was measured in the presence of specific substrates (electron donors) for the single complexes and complex-specific inhibitors after injection of the substance of interest. (A) The cI inhibitor tebufenpyrad was tested in different concentrations regarding its ability to inhibit the MRC complexes I-IV. The cI inhibitor fenpyroximate (10 µM) (B), the cII inhibitor carboxin (100 µM) (C), and the cIII inhibitors picoxystrobin and azoxystrobin (10 µM, 100 µM) (D, E) were tested in single concentrations. (B-E) Black bars indicate the main affected complex. Gray bars indicate apparent inhibition as indirect effect, induced by the inhibition of a subsequent complex. White bars are considered as no inhibitory effect since they did not exceed the threshold. The non-significant range of the assay was defined by 2×SD of the baseline variation and is indicated by the light gray area. Data are means ± SD of three replicates and are given as % inhibition compared to the OCR of control cells (0% inhibition).

A

Target	Substance	Neurite specificity BMC$_{25}$ ratio (V/NA) Glc	Gal
cI	Rotenone	96	10
	Piericidine-A	17	130
	Tolfenpyrad	12	33
	Fenpyroximate	6.6	7.9
	MPP$^+$	2.9	35
	Berberine	1.9	4.5
	Tebufenpyrad	1.6	7.1
	Deguelin	n.t.	7.9
cII	Atpenin A5	910	78
	Isoflucypram	9	38
	Thifluzamide	3.6	6.8
	Bixafen	2.5	20
	Penthiopyrad	1.3	6.0
	Isopyrazam	2.2	2.9
	Mepronil	1.5	2.5
	Carboxine	n.t.	2.6
	3-Nitropropionic acid	n.t.	2.0
cIII	Picoxystrobin	5.5	4.3
	Trifloxystrobin	4.7	3.1
	Azoxystrobin	3.4	4.4
	Pyraclostrobin	2.5	3.5
	Fluoxastrobin	2.1	4.3
	Fenamidone	2.0	3.8
	Kresoxim-methyl	1.7	3.9
	Cyazofamid	1.0	3.6
	Antimycin A	n.t.	2.7
cV	1-Octylguanidine	6.4	5.2
	Oligomycin	n.t.	3.5
uncoupler	Tralopyril	4.6	3.0
	FCCP	3.3	2.9
	2,4-Dinitrophenol	1.1	2.3
others	Colchicine	15	69
	Taxol	120	67
	Chlorfenapyr	1.6	2.3
	Bortezomib	86	340
	Delanzomib	12.0	900
	MG-132	4.2	84
	Epoxomicin	1.8	1.9
	Carfilzomib	1.6	91

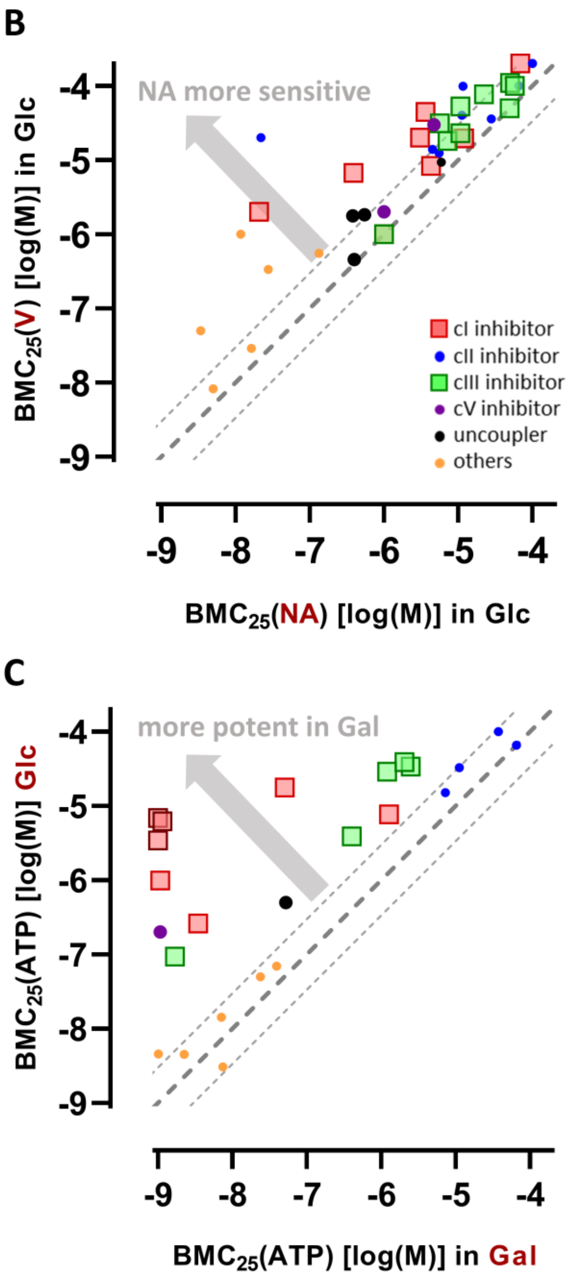

Supplementary Figure S10: Comprehensive overview of tested substances revealing increased specificity of the PeriTox-M assay for neurotoxicants.

The PeriTox and PeriTox-M assays were evaluated regarding their capability to identify neurotoxicants. (A) According to the prediction model, the ratio of the BMC$_{25}$(V) and the BMC$_{25}$(NA), i.e., the concentration at which unspecific cell death occurred versus the concentration at which specific neurite effects were detected, was calculated for all 39 compounds. If this ratio was ≥3, the cells were colored green, and the respective compounds were considered to be specific neurotoxicants. A ratio <3 indicates unspecific cytotoxicity of a compound (red). White cells indicate that no toxicity (n.t.) was detected. (B) The PeriTox assay's capability to detect specific neurotoxic effects in glucose (Glc) medium is illustrated graphically across all tested compounds. The BMC$_{25}$(NA) is given on the x-axis, and the

BMC_{25}(V) on the y-axis. The gray dashed line indicates a BMC_{25}(V)/BMC_{25}(NA) ratio of 1, while the dotted lines indicate a ratio of 3 (and 0.33). Every compound found left of the upper dotted line decreased the neurite area (NA) more potently than the general cell viability. Such compounds were considered specific neurotoxicants. (C) For the 24 compounds that were assessed for their effects on the intracellular ATP levels, the enhancement in sensitivity by Gal is displayed graphically. The BMC_{25}(ATP) in Gal is given on the x-axis, and the BMC_{25}(ATP) in Glc on the y-axis. The gray dashed line indicates a BMC_{25}(NA) [Glc/Gal] ratio of 1, while the dotted lines indicate a ratio of 3 (and 0.33). Every compound found left of the upper dotted line was detected in the PeriTox-M assay with strongly increased sensitivity, indicated by the gray arrow. (B,C) The different compound groups are assigned to shapes and colors. Detailed data are found in supplementary materials, ref. [2].

		Cytotoxicity		Sensitivity ratio
Target	Substance	UKN4b	UKN5b	UKN4b vs UKN5b
cI	**Fenpyroximate**			1.9
	**Berberine**			1.9
	**Deguelin**			1.7
	**Rotenone**			1.0
	**Tebufenpyrad**			0.2
	**MPP+**			0.03
	**Tolfenpyrad**			0.02
cII	**Isopyrazam**			0.9
	**Thifluzamide**			1.3
	**Bixafen**			2.1
	**Isoflucypram**			3.8
	**Atpenin A5**			4.2
	**Penthiopyrad**	x		3.4 *
	**Carboxine**		x	8.6
	**Mepronil**	x	x	1.8
cIII	**Cyazofamid**			1.6
	**Fenamidone**			1.5
	**Azoxystrobin**			1.4
	**Pyraclostrobin**			0.9
	**Picoxystrobin**	x		3.3 *
	**Trifloxystrobin**	x		1.7 *
	**Kresoxim-methyl**	x		1.2 *
	**Antimycin A**		x	0.5
cV	**1-Octylguanidin**			0.4
	**Oligomycin**	x		1.6 *
uncouplers	**FCCP**	x	x	1.7
	**2,4-Dinitrophenol**	x	x	0.8
others	**Colchicine**			6.4
	**Bortezomib**	x		1.1 *
	**MG-132**	x		0.6 *

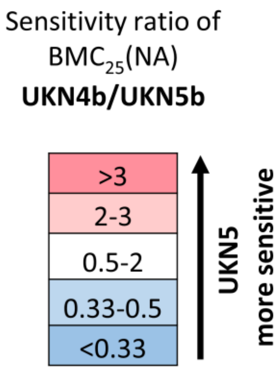

Supplementary Figure S11: Comparison of PeriTox-M effect concentrations to data from a central neuron-based test method.

The PeriTox-M assay (UKN5b) and the UKN4b assay, i.e., the Gal version of the NeuriTox assay (using central neurons; [5]), were evaluated regarding their sensitivity towards effects on the neurite area (NA). BMC_{25}(NA) data of the PeriTox-M assay were compared to UKN4b data from Delp et al. (2019) and Alimohammadi et al. (2023) [5, 6], and the ratio of UKN4b/UKN5b is given in the right column. Red coloring of cells indicates that the UKN5b assay was more sensitive than the UKN4b assay, blue coloring indicates an increased sensitivity of the UKN4b assay. The middle columns show, whether a compound was classified as cytotoxic (x) in one of the assays. For these substances, the interpretation of the BMC_{25}(NA) [UKN4b/UKN5b] ratio is less informative, as the neurite toxicity was an indirect consequence of general cytotoxicity. * The UKN5b assay was more sensitive to detect specific neurotoxicity, as the compound was generally cytotoxic in UKN4b, but specifically neuro-toxic in UKN5b (PeriTox-M).

Supplementary References

1. OECD. "Initial Recommendations on Evaluation of Data from the Developmental Neurotoxicity (Dnt) in-Vitro Testing Battery." In *Oecd Series on Testing and Assessment, No. 377*. Paris: OECD Publishing, 2023, doi:10.1787/91964ef3-en.
2. Holzer, A.-K.; Leist, M. PeriTox-M, a Cell Based Assay for Peripheral Neurotoxicity with Improved Sensitivity for Mitochondrial Inhibitors—Supplementary Data. Zenodo 2025. https://doi.org/10.5281/zenodo.17457804
3. Schildknecht, S., Pape, R., Meiser, J., Karreman, C., Strittmatter, T., Odermatt, M., Cirri, E., Friemel, A., Ringwald, M., Pasquarelli, N., Ferger, B., Brunner, T., Marx, A., Möller, H. M., Hiller, K., and Leist, M. "Preferential Extracellular Generation of the Active Parkinsonian Toxin Mpp+ by Transporter-Independent Export of the Intermediate Mpdp+." *Antioxid. Redox Signal.* (2015) 23, 1001-16, doi:10.1089/ars.2015.6297.
4. Terron, A., Bal-Price, A., Paini, A., Monnet-Tschudi, F., Hougaard Bennekou, S., Angeli, K., Fritsche, E., Mantovani, A., Viviani, B., Leist, M., Schildknecht, S., and Members, Efsa Wg Epi. "An Adverse Outcome Pathway for Parkinsonian Motor Deficits Associated with Mitochondrial Complex I Inhibition." *Arch. Toxicol.* (2018) 92, 41-82, doi:10.1007/s00204-017-2133-4.
5. Delp, J., Funke, M., Rudolf, F., Cediel, A., Hougaard Bennekou, S., van der Stel, W., Carta, G., Jennings, P., Toma, C., Gardner, I., van de Water, B., Forsby, A., and Leist, M. "Development of a Neurotoxicity Assay That Is Tuned to Detect Mitochondrial Toxicants." *Arch. Toxicol.* (2019) 93, 1585-608, doi:10.1007/s00204-019-02473-y.
6. Alimohammadi, M., Meyburg, B., Ückert, A.-K., Holzer, A.-K., and Leist, Marcel. "Efsa Pilot Project on New Approach Methodologies (Nams) for Tebufenpyrad Risk Assessment. Part 2. Hazard Characterisation and Identification of the Reference Point." *EFSA support. publ.* (2023) 20, 7794E, doi:10.2903/sp.efsa.2023.EN-7794.
